# The effect of different carbon materials’ addition on the biomethane production from food waste

**DOI:** 10.1038/s41598-025-02564-0

**Published:** 2025-05-28

**Authors:** Michał Kozłowski, Bernard Papaj, Karolina Sobieraj, Kacper Świechowski, Katarzyna Kosiorowska, Andrzej Białowiec

**Affiliations:** Department of Applied Bioeconomy, Wroclaw University of Environmental and Life Sciences, 51-630 Wroclaw, Poland

**Keywords:** Anaerobic digestion, Torrefaction product, Biochar, Hydrochar, Specific surface area, Biogas, Environmental biotechnology

## Abstract

Anaerobic digestion (AD) is a useful process that could be utilized for food waste (FW) management. Previous studies have shown that carbon materials (CMs) could be an important additive for increasing biomethane yield. However, why CMs improve AD is still uncertain. A significant body of research has been dedicated to investigating the impact of CMs supplementation on biogas production. However, this article specifically emphasizes examining this effect concerning the specific surface area and the functional groups (e.g. hydroxyl groups, carbonyl groups, or unsaturated carbon structures) present on the surface of CMs, produced by torrefaction—TP (240 °C/60 min), pyrolysis—BC (600 °C/60 min), and hydrothermal carbonization—HC (240 °C/60 min/6–10 Bar) processes. The analyses showed that the size of the specific surface area of the CMs (TP—7.72 m^2^ g^−1^, BC- 115.00 m^2^ g^−1^, HC—5.46 m^2^ g^−1^), does not correspond to the production of biomethane. The highest biomethane potential was found for CMs with the lowest SSA, precisely TP and HC, equal to 407 and 394 mL gVS^−1^, which was about 13 and 9% higher than production from FW as a sole source of carbon, respectively. The FTIR analysis confirmed the abundance of different organic functional groups on the surface of TP and HC, which could contribute to improved AD performance. These organic residuals, as thermal degradation products, could be an additional source of carbon for microorganisms. The addition of BC, with the highest SSA, decreased the first-order biomethane rate constant k by 16.4% in comparison to food waste without CMs, which could be related to the presence of harmful, more complex organic compounds on the surface of biochar.

**Supplementary Information:**

The online version contains supplementary material available at 10.1038/s41598-025-02564-0.

## Introduction

One of the most beneficial techniques for reducing pollution and recovering energy from organic waste streams is anaerobic digestion (AD). Although it is a well-established technology, the viability of current AD applications has been improved by the development of many intensification strategies^1^. Various additives have been used to increase the production of biomethane, including microbes, enzymes, biological metabolites, defoaming agents, activated carbon, graphite, plastic carriers, chelating agents, nanoparticles, and diatomite^2^.

Carbon-based materials (CMs) have recently been researched as a potential new additive to increase AD efficiency^3^. Within this group, biochar (BC), a carbonated solid produced by thermochemically converting biomass in an oxygen-depleted atmosphere^4^, has grown in significance. A variety of thermochemical processes can be used to produce CMs, including pyrolysis, torrefaction, and hydrothermal carbonization (HTC). Pyrolysis is carried out at temperatures between 400 and 800 °C under an inert atmosphere, while mild pyrolysis form—with temperatures between 200 and 300 °C—is known as torrefaction^5^. The HTC process transforms the wet biomass to CMs (called hydrochar) at temperatures 170–280 °C under an autogenic pressure of 2–5 MPa^6^.

Beneficial physicochemical properties of CMs, such as large porosity and surface area, are proposed to be the primary drivers of their useful properties as porous structures could create a niche that supports the growth of microorganisms or contains functional groups^7^. It has been suggested that CMs surface properties also have an impact on functional microorganisms’ direct interspecies electron transfer (DIET)^8^. They are seen as the most crucial factors that determine the quantity and quality of active sites present in CMs, improving their cation exchange, water-holding, and adsorption capacities^9^. In CMs structure, macropores facilitate substance diffusion, while mesopores serve as mass transfer routes and micropores as space for trapping^10^. As a result, CMs can shorten the lag phase, improve the maximum methane production rate, and increase the cumulative methane production during AD^1,11,12^. A large specific surface area of CMs could serve as a crucial factor in the removal of pollutants^13^.

The operating parameters of thermochemical CMs production processes can affect the final CMs structure. The physical and chemical characteristics of CMs can be modified by adjusting variables like heating rate, residence time, temperature, gas carrier, and vapor residence time^14^. But above all, the type of process used—pyrolysis, torrefaction, or HTC—significantly affects the characteristics of the final product. For instance, when compared to biochar produced at the same operating process temperature, hydrochar is reported to have a higher heating value and a lower content of heavy metals, alkali, and alkaline earth elements^15^. On the other hand, the hydrochar is characterized by a poor, slightly porous surface and porosity^16,17^. Biochar, a product of the pyrolysis process, is composed of graphite-like layers with surface areas between layers increasing with the growing process temperature. Inversely, the hydrochar surface is built of spherically shaped carbonaceous nanoparticles^15,18^. Additionally, the hydrochar shows a high degree of aromatization on its surface with many oxygen-containing functional groups^15^. The primary explanation of the differences between structural and surface characteristics of BC and hydrochar is the different reaction media employed in the pyrolysis and HTC processes, respectively, which result in different reaction pathways and intermediate compounds^15^.

Since the method of producing CMs from biomass affects their structure and characteristics, and CMs produced in various thermochemical processes differ in porosity and surface area, an important research question is which CMs have the most beneficial effect on the biomethane production process. However, different technological conditions may also lead to the formation of organic matter thermal degradation products, which may condense on the surface of the CMs. These two phenomena may interact. Condensing organic compounds may decrease the SSA by filling the void of the pores. Therefore, we derived the hypothesis that the SSA of CMs’ influence on biomethane production may be diverted by the presence of the organic compounds and residuals after the thermal treatment. Therefore, we checked three types of CMs: biochar (BC) produced at 600 °C and 60 min with high SSA, torrefaction product (TP) produced at 240 °C for 60 min, and hydrochar (HC) produced at 240 °C for 60 min at 6–10 bar having low SSA, however, characterized by the high abundance of the functional groups. Therefore, the research aimed to compare the impact of the addition of CMs with different specific surface characteristics with different functional groups abundance on the biomethane yield and kinetics in the AD process. The CMs were used as an additive in the laboratory-scale food waste (FW) AD process.

## Results and discussion

### Materials characteristics

#### Ultimate and proximate analysis of the carbonaceous material and wheat straw

The properties of wheat straw, food waste, and the obtained CMs were summarized in Table 1.

**Table 1:** Summarized features of substrates and CMs.

Parameter	Food waste	Wheat straw	TP	BC	HTC
TS, %	23.1	92	99	98,5	30
VS, %_TS_	92.4	97	97	91	96
MC, %	76.9	8	1	1.5	70
AC, %_TS_	7.6	2.9	3.1	9.2	4.0
HHV, J/g_TS_	–	18,548	20,054	30,892	24,115
pH	–	6.8	6.0	10.5	5.5
SSA, m^2^/g	–	20.03	7.72	115	5.46
C, %_TS_	44.6	45.6	50.2	86.2	66.2
H, %_TS_	6.2	6.4	5.4	2.2	5.1
N, %_TS_	4.0	0.7	0.6	0.9	0.9
S, %_TS_	1.7	1.7	1.4	0.7	1.3
O, %_TS_	35.9	42.7	39.3	0.8	22.5

Biochar had the highest pH (10.5), while the rest of the CMs had lower pH values than the straw pH (6.8) from which they were made. The most significant difference in pH was observed between biochar and hydrochar pH (5.5). The highest observed pH present in biochar samples may be associated with the formation of carbonates (which is promoted at elevated temperatures^19,20^. However, the pyrolysis process could remove volatile solids with leftovers with a high Ph^19,21^. On the other hand, the lower pH observed in the torrefacation product and hydrochar is related to the decomposition of acidic compounds, which led to the formation and release of volatile organic acid^22^. The low pH of the hydrochar can be attributed to the temperature at which the material was produced (240 °C) and the use of wheat straw as the feedstock. Wheat straw, which contains a significant amount of cellulose, may undergo degradation (at temperatures exceeding 200 °C), leading to the formation of organic acids. These acids could contribute to the acidification of the hydrochar, thereby reducing its pH^23^. In the case of hydrochar, where the pH was the lowest, unlike biochar, which possesses the ability to retain or accumulate alkaline minerals during the pyrolysis process, hydrochar production can result in the loss of these components. This is particularly evident when the raw material is processed under conditions promoting alkaline minerals release, resulting in a product with reduced alkalinity and lower pH^19,24^.

The highest value of HHV was found for biochar. The results indicate that the HHV increased for each CMs, which is consistent with the previous literature^25,26^. During the HTC process, the HHV was higher than raw feedstock compounds as mentioned in^15^.

Results for volatile solids content show that the highest value was characterized by TP and HC, similar to wheat straw (Table 1). This is similar to Tag et al.^27^ and Zhang et al.^28^ findings, where hydrochar had more VS content than biochar. Food waste VS content was high, more than 92% of dry solids, showing it had good applicability to AD.

The results of tests of the specific surface area of carbon materials and wheat straw are presented in Table 1. They show that the largest surface area is characteristic of biochar produced in the pyrolysis process (115 m^2^ g^−1^). Despite the torrefaction process (7.7 m^2^ g^−1^) and hydrothermal carbonization (5.5 m^2^ g^−1^), the results show that the starting material (wheat straw) has a larger surface area (20 m^2^ g^−1^) than these two CMs. Torrefaction and HTC processes did not increase the specific surface area, which could be associated with the thermal degradation products condensed on the surface of these CMs, which was confirmed by FTIR analysis, showing the higher abundance of the different functional groups on TP and HC. Biochar had a larger specific surface area than TP and HC, which was also confirmed by Dieguez-Alonso et al.^29^, and Wang et al.^30^.

#### FTIR analysis results for carbonaceous materials and wheat straw

**Fig. 1:**
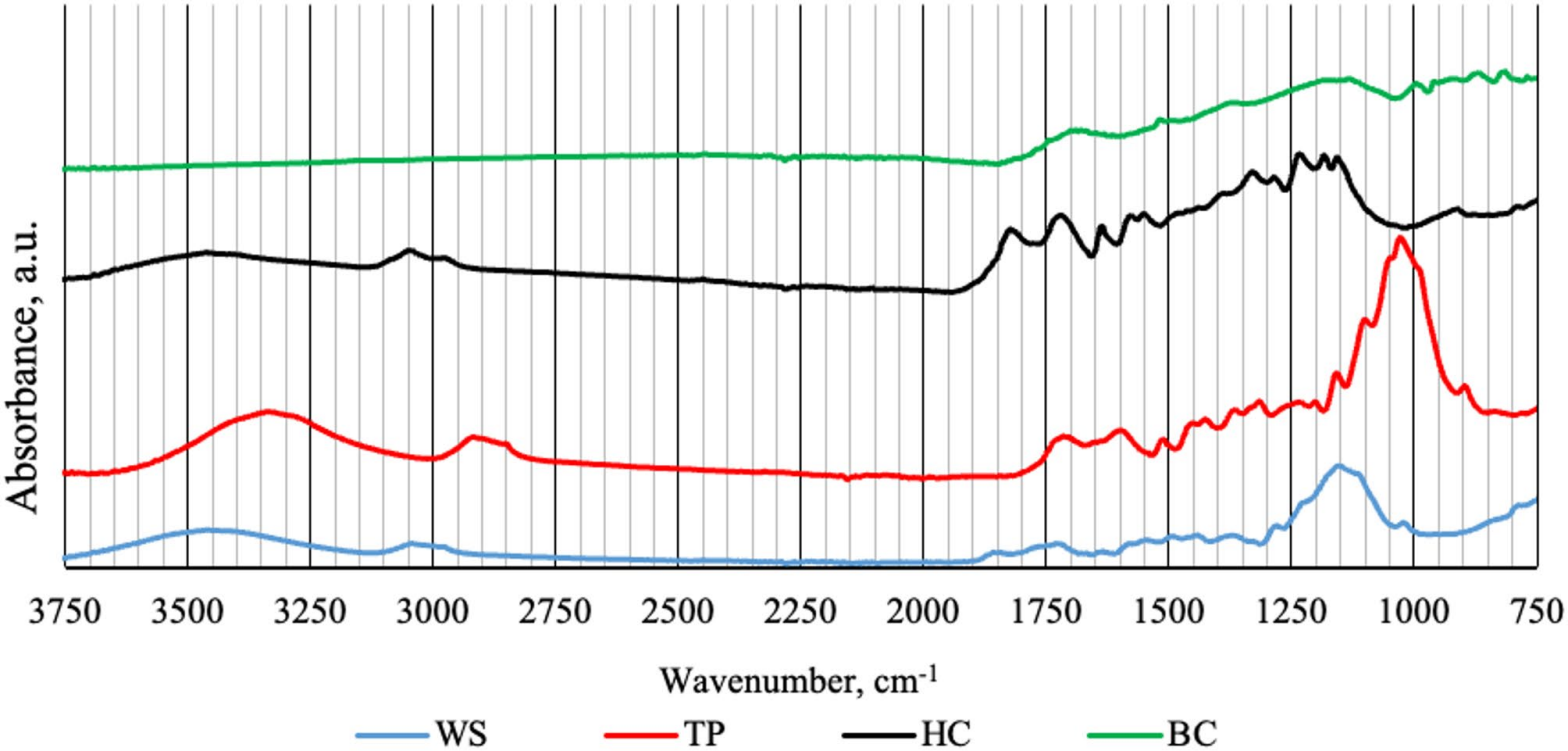
FTIR spectra of wheat straw and CMs. WS, wheat straw; TP, torrefaction product; HC, hydrochar; BC, biochar.

FTIR spectroscopy is highly effective in detecting the shifting of functional groups in studied materials. Figure 1 shows the FTIR spectra of dry wheat straw (WS, blue line), and CMs made of wheat straw as: torrefied product (TP, red line), hydrochar (HC, black line), biochar (BC, green line). Wheat straw is a lignocellulose material that is made of hemicelluloses, cellulose, and lignin. Thus, most of the peaks are considered to come from those components. In the 3750–1900 cm^−1^ range, the WS showed three peaks at 3456, 3028, and 2985 cm^−1^. The peaks in the range of 3700–3100 cm^−1^ indicate the presence of hydroxyl groups (O–H) from water or alcohols, while low-intensity peaks in the range of 3000–2800 cm^−1^ suggest the presence of a small amount of aliphatic carbon–hydrogen (C–H) compounds. Within the 3750–1900 cm^−1^ range, HTC did not change significantly FTIR spectra, while torrefaction resulted in spectra shifts to the right, and pyrolysis resulted in peaks vanishing. It is suggested that the pyrolysis led to complete or almost complete degradation of hydroxyl groups (which are associated with water-containing compounds, such as chemically bound water) and aliphatic structures (mainly hemicelluloses); however, to confirm this, further studies would be required in this regard. In contrast, torrefaction was less intense than pyrolysis, leaving some water-containing compounds and incompletely degraded hemicelluloses. In the range 1900–750 cm^−1^, spectra of wheat straw and CMs become more complex. Interestingly, the wheat straw showed a weak peak at 1850 cm^−1^, which is not typical for this material^31^. It may be the result of sample preparation for the experiment since the sample was dried at 105 °C for 24 h before FTIR. As a result, the temperature could remove bound water and slight dehydration of oxygen-containing functional groups, such as carboxylic acids, leads to the formation of acid anhydrides observed in this range^32^. Generally, in the 1800–1500 cm^−1^ range, wheat straw showed weakly marked peaks. At the same range, some peaks were more intense for torrefied and hydrothermally carbonized wheat straw, while not present in biochar. For lignocellulose materials, the bands between 1740 and 1510 cm^−1^ are mainly related to unsaturated carbon structures (C=C) and carbonyl groups (C=O)^33^. The results indicate that wheat straw had a low content of the mentioned structures, but torrefaction and hydrothermal carbonization increased this content, likely due to the partial decomposition of hemicelluloses and cellulose and the condensation of the products on the surface. The intensity of pyrolysis led to higher decomposition and further conversion into other carbon structures. A similar trend can be observed for the range of 1500–1250 cm^−1^, which is related to the presence of (C–H) and (C–O) functional groups^33^. In the 1250–750 cm^−1^ range can be observed the highest peak for a wheat straw at 1148 cm^−1^ is related to C–O–C stretching^34^ commonly present in structures of cellulose and hemicellulose (oxygen bridges connecting glucose units into long chains). This peak is also visible for torrefied wheat straw but is not present for hydrochar and biochar, suggesting complete degradation of C–O–C structures in those processes.

### Anaerobic digestion

#### Kinetics of the biomethane production process

Theoretical biomethane potential, based on the elemental composition of food waste, was 457.03 mL gVS^−1^. This value has been considered as a reference for the evaluation of AD performance. The ultimate biomethane potential of FW was about 360 mL gVS^−1^, which is about 79% of the theoretical biomethane potential for the same substrate. More than 20% of the substrate remained unutilized, which may suggest that a portion was consumed for microbiota growth in the solution. Additionally, this could indicate the presence of refractory organic compounds or strongly chemically bound substances, impeding their degradation, within the time of the experiment. The addition of biochar did not improve the ultimate biomethane potential, which consisted of about 363 mL gVS^−1^, however, the application of TP and HC significantly increased the ultimate biomethane potential to 407 and 394 mL gVS^−1^, respectively. TP and HC improved AD efficiency to 89% and 86% of the theoretical biomethane potential of FW, respectively. The results show that these variants had higher yields than the FW alone without additives^35,36^. Although in the literature^37^, a large specific surface area is crucial for biogas yield, the obtained results show that other parameters improve methane production. The CMs with the lowest SSA improved ultimate biomethane potential the most significantly. It could be caused by the presence of the thermal degradation products condensed on the surface of these CMs, which are an additional source of carbon for microorganisms. CMs themselves may contain organic particles that are degradable, which could also have an impact on the enhancement of biogas production when supplemented with CMs. The richness of the organic functional groups on the surface of TP and HC, contrary to BC, was confirmed by FTIR analysis. The data related to the kinetic parameters of biogas production are provided in Table 2.

**Table 2:** Biomethane production kinetics.

Parameter	Control	Control + FW	Control + FW + TP	Control + FW + BC	Control + FW + HC
B_0_, mL gVS^−1^	57.84 ± 4.55	360.53 ± 14.73	406.62 ± 2.38	362.68 ± 28.74	393.62 ± 1,72
k, d − 1	0.218 ± 0.013	0.244 ± 0.015	0.218 ± 0.000	0.204 ± 0.022	0.221 ± 0.004
r, mL gVS^−1^ d^−1^	12.62 ± 1.69	87.92 ± 1.60	88.53 ± 0.34	74.52 ± 13.56	87.13 ± 1.18
t0.5, d	3.19 ± 0.19	2.84 ± 0.17	3.18 ± 0.01	3.42 ± 0.40	3.13 ± 0.05
R^2^	0.982 ± 0.003	0.954 ± 0.005	0.966 ± 0.000	0.946 ± 0.018	0.961 ± 0.002

The highest value of ultimate biomethane potential for a combination of Control + FW + TP indicates that substrate combinations with the addition of TP can lead to synergistic effects, increasing the total biomethane production^38^. In the case of analysis of ANOVA (Table 3), it was observed that variants: Control + FW, Control + FW_TP, Control + FW + BC, and Control + FW + HC, achieved statistically significant differences (*p* < 0.05) regarding the maximum cumulative volume of biomethane compared to the control sample. There were no significant statistical differences between variants FW and FW with every CMs additive. However, the value of *p* = 0.057 for the combination Control + FW + TP vs Control + FW shows that the significance difference could be confirmed if the confidence level increased to *p* < 0.1.

**Table 3:** ANOVA test results for the B_0_ variable, fields marked in red are statistically significant (*p* < 0.05).

	Tukey Test; variable: B_0_) The marked values are statistically significant *p* < 0.05				
	Control M = 57.836	Control + FW M = 360.53	Control + FW + TP M = 406.62	Control + FW + BC M = 362.68	Control + FW + HC M = 393.62
Control		0.000180	0.000180	0.000180	0.000180
Control + FW	0.000180		0.056976	0.999779	0.145288
Control + FW + TP	0.000180	0.056976		0.071218	0.880840
Control + FW + BC	0.000180	0.999779	0.071218		0.184653
Control + FW + HC	0.000180	0.145288	0.880840	0.184653	

In the work of Quintana-Najera et al.^39^, a statistical difference was confirmed only between the sample with substrate and with substrate with additives. However, Ayodele et al.^40^ showed that the incorporation of hydrochar enhanced biogas production and significantly increased methane concentration, attributed to its high ash content containing elements such as magnesium, potassium, and phosphorus, which facilitate carbon dioxide capture. The biochemical methane potential (BMP) of hydrochar itself was not statistically significant.

**Fig. 2:**
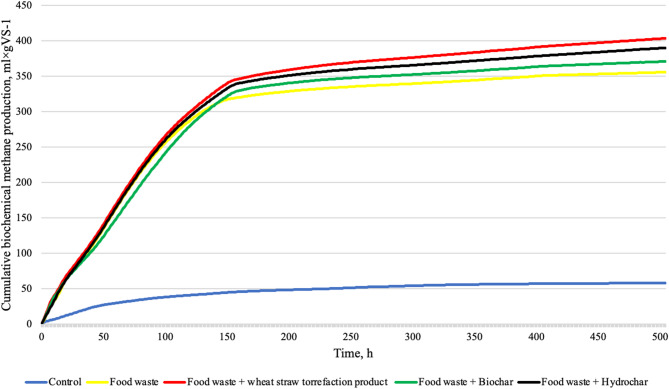
The average cumulative biochemical methane production.

Figure 2 shows the average values of cumulative biomethane production. It can be seen that intense biomethane production occurred during the first 150 h, after which the biomethane production decreased. However, 50% of the biomethane production was achieved after a time between 2.8 and 3.4 days, showing the high biodegradability of the substrate. The highest yields were obtained with reactors with TP and HC additives. This indicates the positive impact of these CMs on biomethane production^41^.

The addition of CMs influenced the first-order biomethane rate constant. K value in TP, BC, and HC variants decreased in comparison to the FW variant by 10.7, 16.4, and 9.4%, respectively. In the case of the combination of variants Control + FW vs. Control + FW + BC, a statistically significant (*p* < 0.05) decrease of k value could be observed when BC was added (Table 5), which may indicate a factor inhibiting the microbial activity. This factor could be related to the presence of numerous harmful volatile organic compounds on the BC surface^42,43^. A similar observation of the negative impact of biochar on k value was observed by Valentin et al.^44^. However, Świechowski et al.^41^, Valentin & Białowiec^45^ or Shen et al.^46^ observed the positive influence of biochars on the first-order biomethane rate constant. Due to inconsistency among different research and the potentially harmful influence of CMs on biomethane formation^47^, further systematic studies are required. The results of the ANOVA test for the k variable are presented in Table 4, and the ANOVA results corresponding to the r parameter are presented in Table 5.

**Table 4:** ANOVA test results for the K variable, fields marked in red are statistically significant (*p* < 0.05).

	Tukey Test; variable: k) The marked values are statistically significant *p* < 0.05				
	Control M = 0.218	Control + FW M = 0.244	Control + FW + TP M = 0.218	Control + FW + BC M = 0.204	Control + FW + HC M = 0.221
Control		0.217781	1.000000	0.775064	0.996753
Control + FW	0.217781		0.307466	0.041466	0.337514
Control + FW + TP	1.000000	0.307466		0.827248	0.998291
Control + FW + BC	0.775064	0.041466	0.827248		0.591455
Control + FW + HC	0.996753	0.337514	0.998291	0.591455	

**Table 5:** ANOVA test results for the R variable, fields marked in red, are statistically significant (*p* < 0.05).

	Tukey Test; variable: r) The marked values are statistically significant *p* < 0.05				
	Control M = 12.619	Control + FW M = 87.918	Control + FW + TP M = 88.27	Control + FW + BC M = 74.523	Control + FW + HC M = 87.132
Control		0.000180	0.000180	0.000181	0.000180
Control + FW	0.000180		0.999971	0.169288	0.999875
Control + FW + TP	0.000180	0.999971		0.212145	0.999225
Control + FW + BC	0.000181	0.169288	0.212145		0.207670
Control + FW + HC	0.000180	0.999875	0.999225	0.207670	

The biomethane production rate, r (mL gVS d^−1^), for all reactors except for the control samples ranged from 2.7 to 2.9 mL gVS d^−1^ (Table 3). This indicates that the addition of CMs doesn’t lead to faster biomethane production, and in the case of BC, decreases this phenomenon. The ANOVA test (Table 6) showed significant statistical differences between the FW and FW + CMs variants and the control sample. No statistical difference was observed between the variants with the addition (FW) and with the addition (FW) and CMs. Wei et al. and Li et al. showed that adding biochar could increase the biomethane production rate^48,49^. Guo et al.^50^ showed that adding hydrochar positively impacts the biomethane production rate, but the effect depends on the dose^50^. Research conducted by Liu et al. showed that the addition of biochar made from wheat straw obtained at a temperature of 600 °C for 2 h increased the cumulative methane yield by 35.45%^51^. Krushna Bhujbal et al. showed that biochar, applied with corn stover as the substrate obtained under conditions of 600 °C for 60 min, demonstrated a 37% increase in biogas production compared to the control^52^.

**Table 6:** Percentage, quantity, and features of food waste in the mix of used substrate.

Substrate	FW share (fresh), %	FW share (dry), g	FW share (fresh), g	Moisture, %	Total solid, %	Volatile solids, %	Ash content, %
Orange	1.42	3.67	24.14	84.7	15.3	90.7	9.3
Banana	4.51	8.67	76.67	83.0	17.0	88.2	11.8
Apple	2.58	7.33	43.86	86.7	13.3	96.5	3.5
Lemon	0.55	1.33	9.35	84.4	15.6	92.9	7.1
Potatoes	26.11	24.33	443.87	82.3	17.7	88.1	11.9
Onion	1.41	4.67	23.97	82.4	17.6	70.3	29.7
Lettuce	0.48	3.33	8.16	92.6	7,0.4	72.2	27.8
Cabbage	0.72	3.33	12.24	85.1	14.9	90.8	9.2
Tomato	0.32	2.33	5.44	95.6	4.4	80.0	20.0
Rice	14.55	6.00	247.35	77.9	22.1	99.5	0.5
Pasta	14.84	6.00	252.28	67.9	32.1	99.1	0.9
Bread	6.50	3.00	110.5	34.5	65.5	96.6	3.4
Meat	2.53	3.00	43.01	75.1	24.9	93.5	6.5
Fish	6.12	12.00	104.04	81.1	18.9	94.2	5.8
Cheese	17.37	11.00	295.29	44.4	55.6	92.3	7.7
Cumulatively	100	100	1700	76.9	23.1	92.4	7.6

#### Analysis of volatile fatty acid (VFA)

The volatile fatty acids (VFAs), including acetic, propionic, and butyric acids, are produced during the hydrolysis and fermentation stages of anaerobic digestion and are essential for the subsequent methanogenesis phase, where they undergo further stages of methanogenesis, ultimately being converted into methane and carbon dioxide^53^. In this study, the VFAs content in samples taken after 21 days of the experiment was analyzed to assess the concentration of VFAs in the final stage of the process. The HPLC analysis conducted showed that short-chain carboxylic acids such as formic, acetic, isobutyric, and butyric acids are not present in the digestates. Only propionic acid was detected, with the highest concentration determined in the control sample at 0.0527 ± 0.0001 gL^−1^. VFAs are essential intermediates produced during the decomposition of organic matter, and their concentration may represent the stability and efficiency of the fermentation process. The absence of VFAs observed may suggest that the fermentation process has reached a state of equilibrium or that the dynamics of the microbial community have changed unfavorably. Factors such as pH fluctuations, toxic substances, or nutrient imbalances can adversely affect microbial populations involved in VFAs production^54^. Furthermore, when the organic loading ratio is set excessively high, it can overwhelm the microbial community, leading to rapid acidification and, subsequently, the potential decrease in VFAs production. When the organic loading ratio is high (in this case, 4 kgVS/m^3^·d), it can lead to a reduction in the pH of the solution and the accumulation of VFAs, such as acetic and propionic acids, thereby diminishing the efficiency of biogas production^55^. The compilation of the obtained concentration results with biomethane production indicates that the main reason for their absence was the complete conversion of these acids into methane and carbon dioxide by methanogenic archaea. Studies prove that when the anaerobic digestion process proceeds optimally, methanogens effectively utilize the VFAs formed during acidogenesis, leading to a decrease in their concentration, nevertheless, excessively high concentrations of VFAs synthesized within a short timeframe may result in the inhibition of biomethane production^56^. This phenomenon is often accompanied by an increase in biomethane production, as observed in this paper and previous studies^57^.

## Conclusions

Carbon materials obtained from torrefaction and hydrothermal carbonization increased biomethane production from food waste. Results obtained in the present study indicated that the addition of a torrefaction product may increase the biomethane yield from 79% of the theoretical potential to almost 90% of the theoretical potential.

The research showed that CMs with the lowest specific surface area, such as TP and HC, improved biomethane potential. It could be caused by the presence on the surface of these CMs of organic compounds as surface functional groups^19^, thermal degradation products, which could be an additional source of carbon available for microorganisms. Functional groups present on the surface of biochar may degrade progressively with increasing pyrolysis temperature^58^. The porous structure of carbon materials may provide niches in which methanogenic microorganisms localize or to which they adhere. The surface pores may offer a more favorable habitat compared to the remainder of the solution^59^, potentially contributing to an increase in biogas production. This effect could also be influenced by the presence of functional groups on the surface, which may be utilized by the microorganisms^59^. The surface of biochar may also adsorb inhibitory compounds that adversely impact microbial growth^2,60^. All CMs decreased the first-order biomethane rate constant k; however, the biochar influenced this parameter to the most significant degree. This phenomenon could be associated with the presence of organic compounds inhibiting microbial activity. Future research should focus on the determination of qualitative and quantitative organic compounds characteristics on the surface of CMs.

## Materials and methods

### Carbon materials production

For the obtaining of CMs, wheat straw harvested in central Europe, Poland, has been used. First, the substrate was gridded with a laboratory knife mill (32,000 RPM, Chemland, Stargard, Poland) to < 1 mm particle size. Obtained wheat straw powder was dried at 105 °C for 24 h in a drying chamber (Wamed KBC-100W, Warsaw, Poland) ground and dried material was used in 3 thermochemical processes to obtain CMs: torrefaction (240 °C for 60 min), pyrolysis (600 °C for 60 min), and hydrothermal carbonization (HTC, 240 °C for 60 min with pressure 6–10 bar), the CMs that were received were named torrefied product (TP), biochar (BC), and hydrochar (HC).

Torrefaction and pyrolysis processes were conducted in a muffle furnace (8.1/1100; SNOL, Utena, Lativa) following^61^. The dry wheat straw powder was placed into a ceramic crucible and put into a muffle furnace chamber. The furnace chamber was sealed and filled with an inert gas (CO_2_) flow of 2.5 dm^3 ^min^−1^ to prevent combustion^62^. Then, the furnace was turned on; inert gas was poured into the chamber throughout the whole process. After the process, the furnace was turned off and left for self-cooling. Temperature was controlled by the furnace settings without a predefined heating time, while hydrothermal carbonization was conducted in the high-temperature, high-pressure reactor (Novoclave, Büchi AG, Uster, Switzerland) following^63^, The reactor was heated at a rate of approximately 10 °C per minute with speed mixers are set to operate at 120 rpm. For hydrochar production, 30 g of wheat straw powder was mixed with 30 g of water and placed into a hydrothermal carbonization reactor. The reactor was sealed tightly and turned on. During the process, the autogenous pressure reached 10 bars. After 60 min, the reactor was turned off and set for self-cooling. When the temperature dropped below 30 °C, the reactor was opened, the process gases were released by the valve, and hydrochar was collected. HC was separated from the liquid phase by vacuum filtration, Subsequently, the sample was dried in a laboratory drying chamber (Wamed KBC-100W, Warsaw, Poland) at 105 °C for 24 h.

### Anaerobic digestion process

Digestate used as inoculum in the AD process was obtained from the agriculture biogas plant (Świdnica, Poland, Central Europe). Digestate was filtered to remove fibers and solid materials and left for degassing for 24 h^64^. The substrate in the AD process was a model mix of FW. The percentage of food waste components was prepared based on^38^. To ensure mix homogeneity, each food type was first manually shredded and dried at 105 °C. Next, the proper mass of each component was weighed and ground to 0.2–0.053 mm. After preparation, the material was dried again and secured in an air-tight, vacuum-packed plastic bag. The proportion of individual food waste within the food waste mixture is depicted in Table 6.

The anaerobic digestion process was performed using the automatic methane potential test system (AMPTS II, BPC Instruments, Sweden). The system consisted of 15 batch reactors placed in a water bath. Each reactor had a volume of 600 mL and was equipped with agitation. The reactors were kept in a water bath at a temperature of 37 °C, with automatic stirrers agitating the solution for 2 min every hour.

To check the influence of different CMs on biomethane production from FW, 5 variants were studied. The first variant was control, inoculum only. The second variant consists of inoculum and FW. The next three variants consist of inoculum, FW, and specific CMs. 400 g of inoculum was placed in each reactor. For variants with substrate and CMs, 4.79 g of dry FW and 2 g of CMs were placed. Each variant was performed in triplicate. The AD process was carried out at 37 °C for 21 days. After the process’s end, the digestate sample was collected and analyzed for volatile fatty acid content (VFAs). VFAs are a group of one of the most crucial chemical compounds during methanogenesis. The elemental composition of the digestate used to determine the substrate dose and CMs was 3,56%_TS_, 62,09%_VS_. Based on this, the inoculum-to-substrate ratio (ISR) was established at 2, allowing for the determination of the CMs and FW doses. The biogas measurements were automatically recorded in the AMPTS system (AMPTS II, BPC Instruments, Sweden), and following the process, the biogas was released.

### Anaerobic digestion performance

To assess the anaerobic performance and effect of added CMs, the theoretical (stoichiometric) biomethane potential and ultimate biomethane production kinetics were determined. The theoretical biomethane potential of FW was calculated using the elemental composition according to the Buswell formula^44^ (1):

1 $${C}_{c}{H}_{h}{O}_{o}{N}_{n}{S}_{s}+\left(c-\frac{h}{4}+\frac{o}{2}+\frac{3n}{4}+\frac{s}{2}\right){H}_{2}O\to \left(\frac{c}{2}-\frac{h}{8}+\frac{o}{4}+\frac{3n}{8}+\frac{s}{4}\right){CH}_{4}++\left(\frac{c}{2}-\frac{h}{8}+\frac{o}{4}+\frac{3n}{8}+\frac{s}{4}\right){CO}_{2}+{nNH}_{3}+{sH}_{2}S$$

The kinetic parameters were determined using a first-order equation (FOE) (Eq. 2). The FOE parameters were determined by estimation of biomethane cumulative biomethane production to a FOE using Statistica software (TIBCO, USA).

2 $$\text{B}={\text{B}}_{0}*(1-{\text{e}}^{-\text{k}*\text{t}})$$

B—cumulative biomethane production at a given time (*t*), mL gVS^−1^.

B_0_—ultimate biomethane potential yield, mL gVS^−1^.

e—mathematical constant = 2.718282.

k—first-order biomethane rate constant, d^−1^.

T—process time, d.

Biomethane production rate, mL gVS d^−1^, was calculated based on Eq. 3:

3 $$r={B}_{0}\cdot k$$

The time of the 50% biomethane production (t_0.5_), d, was determined according to Eq. 4:

4 $${t}_{0.5}=\frac{ln2}{k}$$

### Materials analysis

The wheat straw and CMs were analyzed for total solids (TS), volatile solids (VS), moisture content (MC), ash content (AC), high heating value (HHV), reaction (pH), specific surface area (SSA) functional groups (FTIR) and elemental composition (C, H, N, S, O). The FW mixture was analyzed for MC, TS, VS, and elemental composition (C, H, N, S, O). The anaerobic digestion process residues (digestate) were analyzed for VFA content determination.

The MC, TS, VS, and AC were determined using a muffle furnace (8.1/1100; SNOL, Utena, Lativa) and laboratory dryer Wamed KBC-100W, Warsaw, Poland) following PN-EN ISO 18,134–2:2017–0 and PN-EN 15,169:2011 standards, respectively. The VM was determined using thermogravimetry (Czylok, RST 40 × 200/100, Jastrzębie-Zdrój, Poland) following^65^. Measurements were made in triplicate.

The pH analysis was conducted by mixing every material with water in a ratio of 1–10 followed by shaking for 2 h^66^. The samples were shaken using an orbital shaker (ELMI DOS-20L). The pH in the solution was measured using a pH meter (Elmetron CPC-411). Measurements were made in triplicate and averaged using the arithmetic mean.

For SSA analysis, a sample of about 0.2 g was used. The analysis was carried out using a sorption analyzer (ASAP 2020, Micromeritics, Atlanta, USA), using the volumetric method. Before the measurements, the materials were pre-degassed in a vacuum at 300 °C for 6 h. Then, nitrogen sorption was measured at 77 K. Based on the obtained isotherms, the SSA was determined following Brunauer–Emmett–Teller (BET) theory. It should be noted that the degassing temperature of the sample, which exceeded the production temperature of both hydrochar and the torrefaction product (both materials produced at a temperature of 240 °C), may have influenced their properties, such as specific surface area and the presence or absence of certain organic compounds.

The functional groups were determined using reflection-Fourier transform infrared (FTIR). The measurements were made using a Nicolet iN10 integrated infrared microscope with a Nicolet iZ10 external FT-IR module (Thermo Fischer Scientific, Waltham, MA, USA). For each spectrum, 32 scans were averaged in the mid-IR range of 400–4000 cm^−1^^67^.

The elemental composition was determined using an elemental analyzer (Perkin Elmer, 2400 Series, Waltham, MA, USA) following PN-EN ISO 16,948:2015–07 standard.

For VFA analysis, the samples underwent centrifugation at 10 °C at 4500 rpm for 10 min. Following this, they were diluted with Milli-Q water and recentrifuged at the same temperature for 5 min at 15,000 rpm. Both quantitative and qualitative analyses were conducted using Ultra-High-Performance Liquid Chromatography (U-HPLC) with the UltiMate 3000 System from ThermoFisher Scientific, UK. A standard solution of VFA (Volatile Free Acid Mix, CRM46975, Merck, Poland) was prepared at a final concentration of 10 mM and stored at 4 °C following the manufacturer’s instructions. The standard curve was established by appropriately diluting the analytes in Milli-Q water. Ethanol standards were also obtained from a supplier. The concentration of VFA and other components, including ethanol, was analyzed using a HyperREZ XPCarbohydrateH + 8 μm column (Thermo Scientific, Waltham, MA). Analyte identification was performed using a UV/VIS-DAD detector set at 208 ± 1 nm. A refractive index (RI) detector (Shodex, Ogimachi, Japan) was utilized concurrently to detect the potential presence of ethanol in the samples. The eluent used was 0.25 mM trifluoroacetic acid, with a flow rate of 1.1 mL/min under isocratic elution conditions, and the column temperature was maintained at 35 °C. Data was analyzed using Chromeleon 7.1 Software, with all samples analyzed in triplicate.

### Statistical analysis

The collected data results were used for basic statistical analysis and graphical interpretation using Microsoft Office Excel 2021 and Statistica 13 (TIBCO, USA) software. To obtain the kinetics parameter (B_0_, k, r) FOE has been used with the least squares method and Levenberg–Marquardt estimation method, nonlinear estimation (95% confidence interval, alpha 0.05). ANOVA post-hoc test (Tukey) was used to determine the statistical significance of the differences between individual kinetic parameters.

## Electronic supplementary material

Below is the link to the electronic supplementary material.

Supplementary Material 1

## Data Availability

All data are available in the form of a dataset submitted to the Knowledge Base repository of UPWr. DOI: 10.57755/t5p2-3c25.
